# Low EEG Gamma Entropy and Glucose Hypometabolism After Corpus Callosotomy Predicts Seizure Outcome After Subsequent Surgery

**DOI:** 10.3389/fneur.2022.831126

**Published:** 2022-03-24

**Authors:** Kenzo Kosugi, Keiya Iijima, Suguru Yokosako, Yutaro Takayama, Yuiko Kimura, Yuu Kaneko, Noriko Sumitomo, Takashi Saito, Eiji Nakagawa, Noriko Sato, Masaki Iwasaki

**Affiliations:** ^1^Department of Neurosurgery, National Center Hospital, National Center of Neurology and Psychiatry, Kodaira, Japan; ^2^Department of Child Neurology, National Center Hospital, National Center of Neurology and Psychiatry, Kodaira, Japan; ^3^Department of Radiology, National Center Hospital, National Center of Neurology and Psychiatry, Kodaira, Japan

**Keywords:** corpus callosotomy, FDG-PET, gamma regularity, lateralization, multiscale entropy, seizure outcome

## Abstract

**Background:**

Patients with generalized epilepsy who had lateralized EEG abnormalities after corpus callosotomy (CC) occasionally undergo subsequent surgeries to control intractable epilepsy.

**Objectives:**

This study evaluated retrospectively the combination of EEG multiscale entropy (MSE) and FDG-PET for identifying lateralization of the epileptogenic zone after CC.

**Methods:**

This study included 14 patients with pharmacoresistant epilepsy who underwent curative epilepsy surgery after CC. Interictal scalp EEG and FDG-PET obtained after CC were investigated to determine (1) whether the MSE calculated from the EEG and FDG-PET findings was lateralized to the surgical side, and (2) whether the lateralization was associated with seizure outcomes.

**Results:**

Seizure reduction rate was higher in patients with lateralized findings to the surgical side than those without (MSE: *p* < 0.05, FDG-PET: *p* < 0.05, both: *p* < 0.01). Seizure free rate was higher in patients with lateralized findings in both MSE and FDG-PET than in those without (*p* < 0.05).

**Conclusions:**

This study demonstrated that patients with lateralization of MSE and FDG-PET to the surgical side had better seizure outcomes. The combination of MSE and conventional FDG-PET may help to select surgical candidates for additional surgery after CC with good postoperative seizure outcomes.

## Introduction

Corpus callosotomy (CC) is a palliative treatment, but not cure, for medically intractable epilepsy in patients who are not candidates for resective epilepsy surgery, and is based on the reduction of interhemispheric propagation of epileptic activity. In general, CC has been recommended for patients with drop attacks caused by tonic, atonic, and myoclonic seizures or epileptic spasms. Such episodes are often characterized by generalized or bilaterally synchronized epileptiform discharges detected by electroencephalography (EEG). Routine scalp EEG cannot always distinguish whether generalized epileptiform discharges result from primary bilateral synchrony or secondary bilateral synchrony from a focal origin. Secondary bilateral synchrony is defined as the rapid bilateral spread from a limited cortical area of electrical abnormality (1).

Corpus callosotomy often results in dramatic changes in interictal spikes, such as desynchronization or lateralization of bilateral spikes (2–5). Postcallosotomy lateralization of epileptiform discharges may reveal epileptic foci in one hemisphere, thus providing a secondary guide to further surgery (6–8). However, the chance of seizure freedom is not very high even after such subsequent surgery (8, 9).

Positron emission tomography (PET) with 2-deoxy-2[^18^F]fluoro-D-glucose (FDG) demonstrates interictal focal cortical glucose hypometabolism ipsilateral to the seizure focus in most patients with chronic focal epilepsy. High resolution FDG-PET achieved sensitivity of 92% and specificity of 62.5% for the detection of frontal lobe epileptic foci in a predominantly pediatric population (10). However, positive FDG-PET findings can be found even in patients with poor postoperative seizure outcomes due to the relatively low specificity, so other modalities are needed to select appropriate candidates for subsequent radical surgeries after CC (11, 12).

Analysis of the complexity of EEG signals holds considerable interest. Multiscale entropy (MSE) (13, 14), an information-theoretic index that estimates sample entropy at multiple time scales (15), is a promising method to quantify the irregularity of neural time series across different brain states, lifespans, and the relationship between health and disease (16–22). The low entropy of interictal gamma oscillations (stationary signals or rhythmic fluctuations) might be a biomarker for the seizure onset zone on intracranial EEGs in patients with focal cortical dysplasia, based on the hypothesis that the synchronization of epileptic interneurons could be evaluated based on the regularity of gamma oscillations (23, 24).

We hypothesized that both FDG-PET and gamma entropy analysis would be useful to evaluate the epileptogenic side after CC, and the combination of the two modalities would improve the specificity to predict seizure outcome after subsequent surgery. This study investigated whether MSE combined with FDG-PET could be a novel diagnostic tool to identify lateralization of the epileptic focus after CC and assess indications for subsequent surgery.

## Materials and Methods

### Patients' Characteristics

This retrospective study included 14 patients with pharmacoresistant epilepsy who underwent curative epilepsy surgery subsequent to CC between 2004 and 2018 in our institution. A total of 102 patients received CC as a palliative procedure during the same period.

Comprehensive presurgical evaluation, which includes long-term video-EEG monitoring and high-field magnetic resonance imaging (MRI), was performed in all 14 patients. The surgical indication was determined in the patient management conference attended by board-certified neurosurgeons, pediatric neurologists, and epileptologists. Intracranial EEG study that includes functional mapping was performed when indicated. CC was considered for patients diagnosed with generalized or multifocal epilepsy and meeting the following criteria: (1) semiology of seizures was generalized atonic, tonic, tonic-clonic, myoclonic seizures, or epileptic spasms; (2) bilaterally synchronous or multifocal interictal epileptiform discharges (IEDs) were detected by EEG; and (3) resectable epileptogenic zone could not be estimated from the comprehensive presurgical evaluation. Seizures were not controlled, and IEDs were lateralized to one hemisphere after CC in all 14 patients. Therefore, subsequent surgery was indicated after repeat presurgical evaluation. Seizure outcomes following the second surgery were evaluated using the International League Against Epilepsy (ILAE) outcome scale (25) and seizure reduction rate (SRR) at 1 year after surgery. SRR was defined as follows:


$$\begin{array}{l} \text{Seizure reduction rate }\left(\text{SRR}\right)\\ =\frac{\left(\text{preoperative seizure frequency}\right)-\left(\text{postopeerative seizure frequency}\right)}{\text{preoperative seizure frequency}}\\ \times 100\;\left(\text{\%}\right) \end{array}$$

95% or higher seizure reduction was defined as favorable seizure outcome.

This study was approved by our institutional review boards, and written consent form was waived for the retrospective design.

Table 1 summarizes the patients' characteristics. A number of 6 male and 8 female patients were aged 1 to 37 years (median age at CC 3.8 years). A number of 6 patients with previous history of West syndrome presented no hypsarrhythmia at the time of preoperative EEG. The etiology of epilepsy, according to the ILAE classification (26), was structural in 11 patients, genetic in one, and unknown in 3. The findings of presurgical evaluation are summarized in Supplementary Table 1. A number of nine patients (64%) had abnormal findings on MRI. However, their seizure semiology and EEG findings were multifocal or generalized, and CC was indicated as a palliative treatment. The median interval to subsequent surgery was 21 months (range, 4 to 111 months). Intracranial electrode implantations were performed in 9 patients (64%). The second surgery was focal cortical resection (FCR) in 7 patients (50%), total hemispherotomy in 4 (29%), subtotal hemispherotomy in 2 (14%), and posterior quadrant disconnection in one (7%). In cases where invasive EEGs were performed, subsequent surgery was guided by those findings, and the area of seizure onset was completely removed in all patients. Hemispheric surgery was performed without invasive evaluation in four patients. The complete removal of a tuber was performed without invasive evaluation in one patient (Case 13) with tuberous sclerosis complex. He had multiple tubers in both hemispheres, but a right frontal tuber was suspected as the epileptogenic origin based on preoperative non-invasive evaluations.

**Table 1:** Summary of clinical, EEG multiscale entropy, and FDG-PET findings in 14 epilepsy patients undergoing CC.

**Case**	**Age at CC**	**Sex**	**Previous history of epilepsy syndrome**	**Etiology of epilepsy**	**Lateralization to** **surgical side**		**Interval between the last clinical seizure and the PET scan (h)**	**Interval to subsequent surgery (month)**	**Intracranial electrode implantation**	**Subsequent surgery**	**SRR**	**Seizure outcome (ILAE)**
					MSE^*^	FDG-PET^**^						
1	1 y	F	West syndrome	Unknown	y	y	3	111	+	PQD	100	1a
2	2 y 4 m	F	-	FCD	y	y	1	26	+	FCR	100	1a
3	2 y 9 m	M	West syndrome	FCD	y	y	8	32	+	FCR	100	1a
4	1 y 11 m	M	West syndrome	FCD	y	y	1	9	–	STH	100	1a
5	6 y	M	-	Hemimegalencephaly	y	y	5	18	–	TH	100	1a
6	5 y 11 m	F	-	Rasmussen syndrome	y	y	6	7	+	TH	100	1a
7	2 y 4 m	F	-	Perinatal insult (Ulegyria)	n	y	2	9	+	FCR	95	3
8	10 y	F	West syndrome	Unknown	y	y	4	58	+	FCR	95	4
9	1 y	F	West syndrome	FCD	y	y	3	4	–	TH	95	4
10	1 y 11 m	M	West syndrome	Unknown	n	y	1	36	+	STH	90	4
11	12 y	F	-	FCD	y	y	24	14	+	FCR	75	4
12	14 y	F	-	FCD	n	y	2	54	+	FCR	38	5
13	4 y 10 m	M	-	Tuberous sclerosis complex	n	n	2	13	–	FCR	0	5
14	37 y	M	-	Infection (Ulegyria)	y	n	3	24	–	TH	0	5

* *Judged lateralized when MSE was lower in the surgical side*.

** *Judged lateralized when the hypometabolism was observed in the surgical side. EEG, electroencephalography; FDG-PET, positron emission tomography with 2-deoxy-2[^18^F]fluoro-D-glucose; CC, corpus callosotomy; MSE, multiscale entropy; SRR, seizure reduction rate; ILAE, International League Against Epilepsy; FCD, focal cortical dysplasia; PQD, posterior quadrant disconnection; FCR, focal cortical resection; STH, subtotal hemispherotomy; TH, total hemispherotomy*.

### Data Acquisition and Preparation

Scalp EEG and FDG-PET were obtained 6 months after CC in 13 patients and 3 months after CC in one patient. Subsequent surgery was performed 4 months after CC in the latter case (Case 9).

Electroencephalography data were acquired using a Neurofax EEG 1200 (Nihon-Kohden, Tokyo, Japan). EEG electrodes were placed in accordance with the international 10–20 scalp electrode positions. The sampling rate was set at 1,000 Hz in all patients. Low-cut and high-cut filters were set at 0.3 and 300 Hz. Five one-min epochs during the early stage of non-rapid eye movement sleep were extracted manually, separated by at least 1 h from any seizures. EEG during wakefulness was more likely to be contaminated with electromyography, which could affect EEG analysis; therefore, we extracted only from sleep stage. The EEG epochs were obtained from one night in all except one case. Artifacts such as eye movements were visually identified and excluded. The antiseizure medication was not reduced during EEG in all cases.

FDG-PET was acquired using a combined 16-slice PET–computed tomography (CT) scanner (Biograph 16; Siemens, Erlangen, Germany). After patients had fasted for more than 6 h, their blood glucose levels were measured before the intravenous injection of 4–6 MBq/kg FDG 40 min before the start of the brain PET–CT scan. It was confirmed that the patient had no clinical seizure at least 1 h before the injection of FDG (Table 1).

Electroencephalography data were extracted using the longitudinal bipolar derivation except for the midline 2 channels. The data consisted of EEG signals from a total of 16 channels, which include Fp1-F7, F7-T3, T3-T5, T5-O1, Fp1-F3, F3-C3, C3-P3, and P3-O1 for the left side and Fp2-F8, F8-T4, T4-T6, T6-O2, Fp2-F4, F4-C4, C4-P4, and P4-O2 for the right side. Data were filtered using a 50-Hz notch filter and downsampled to 200 Hz using the EEGLAB toolbox (http://sccn.ucsd.edu/eeglab) to efficiently analyze the gamma frequency ranges. The MSE scores were calculated using sample entropy (27). As described in detail in the previous reports (23, 27), MSE scores with τ (the time scale factor) depend on three parameters: N (the total number of data points), m (the number of the consecutive data points to be compared), and r (a noise threshold for measuring the consistency of the time series). Various theoretical and clinical applications have shown that m = 1 or 2 and r = 0.1–0.25 of the standard deviation of the data points provides good statistical validity for sample entropy (28, 29). Moreover, the previous studies that estimated the sample entropy or the MSE from EEG dataset have shown that values of m = 2 and r=0.2 provide good statistical validation for MSE analysis (17, 23, 24, 30, 31). In this study, for each selected 60-s epoch, we calculated MSE scores with *N* = 12,000 (i.e., 60 s × 200 Hz), m = 2, and r = 0.2. Sato et al. (23) have shown that MSE score for gamma oscillation was lower in seizure onset zone in cases where subdural electrodes were implanted, and therefore, we applied the MSE for gamma oscillation in the scalp EEG settings. Given the sampling rate of 200 Hz, the time scale factor τ=3 to 7 approximately corresponded to the gamma frequency (30–70 Hz), because 200/3 = 66.7 Hz, and 200/7 = 28.6 Hz. The MSE score was calculated for each EEG epoch in each channel using MATLAB_R2019b (MathWorks, Natick, MA, USA). The MSE scores derived from the five EEG epochs were checked for normality using the Shapiro–Wilk normality test and checked for variance using Bartlett's test, and the mean of the five scores was used as the representative MSE value of an EEG channel (Supplementary Figure 1).

### Lateralization to the Surgical Side

Multiscale entropy scores were statistically compared between the left and right hemispheres (8 values on each side) to judge whether the lower MSE score was concordant with the surgical side (Supplementary Figure 1). SRR was compared between patients with and without lateralized MSE scores. The association between the lateralized MSE and seizure freedom was evaluated, and the sensitivity and specificity of the lateralized MSE to the favorable seizure outcome were calculated.

Neuroradiologists have previously interpreted FDG-PET images by visual inspection as a part of the routine clinical evaluation in each patient. According to the interpretation, neurosurgeons and neuroradiologists determined whether the hypometabolic hemisphere, if any, was concordant with the surgical side. The following analysis was the same as above in MSE scores.

Finally, whether both low MSE score and FDG-PET hypometabolism were lateralized to the surgical side was evaluated. The following analysis was the same as above.

### Statistical Analysis

Statistical analyses were all conducted with R version 4.0.0 (The R Foundation for Statistical Computing). MSE scores of the left and right hemispheres were compared using a two-sided paired *t*-test. The difference in SRR between the two groups was examined with the Wilcoxon rank sum test. The association of seizure freedom was evaluated by Fisher's exact test. *p*-Values of <0.05 were considered statistically significant.

## Results

**Figure 1:**
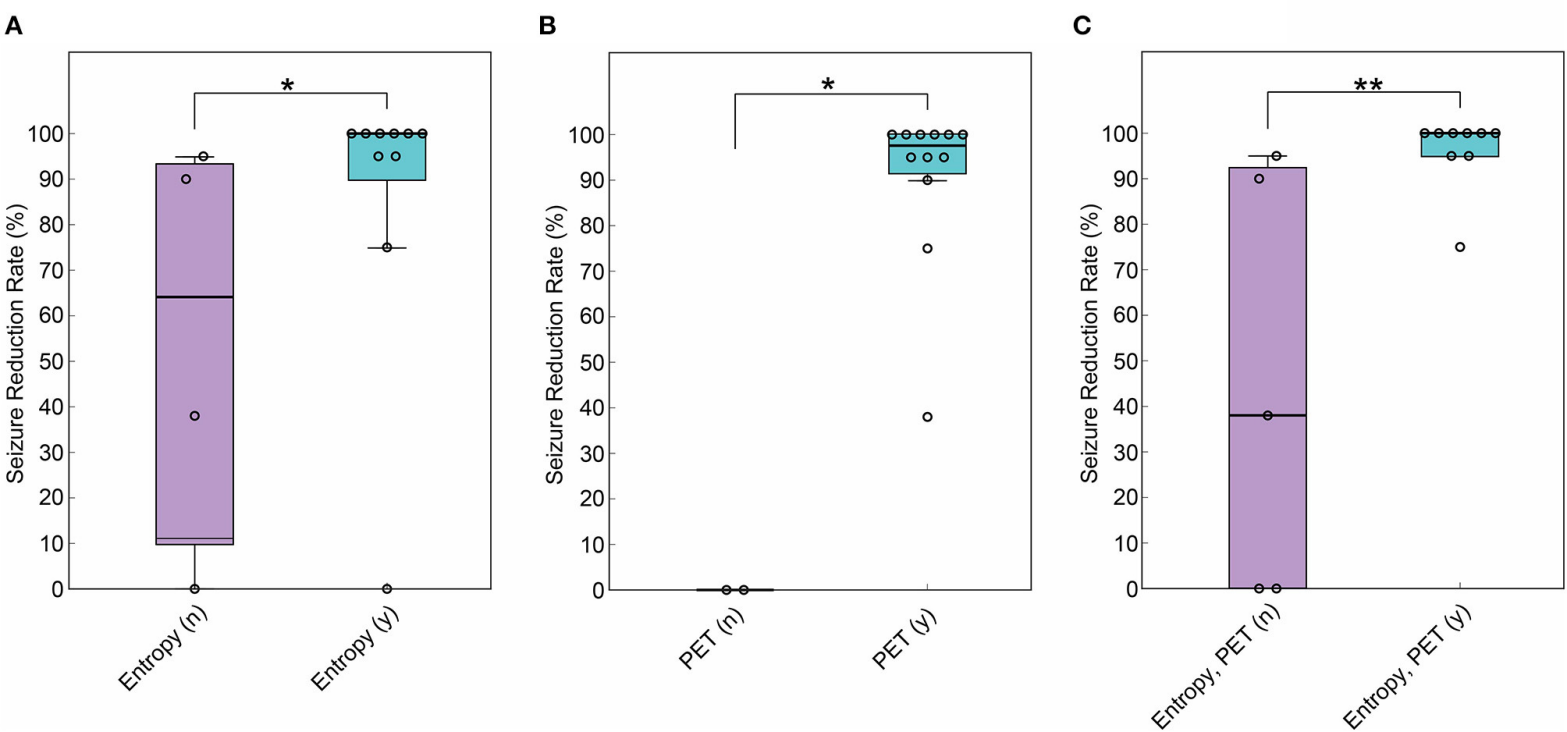
Comparison of SRR between patients with and without lateralization of gamma entropy and positron emission tomography with 2-deoxy-2[18F]fluoro-D-glucose (FDG-PET) findings to the surgical side. **(A)** SRR was higher in the patients with multiscale entropy (MSE) showing lateralization to the surgical side (*n* = 14, Wilcoxon rank sum test, *p* < 0.05). **(B)** SRR was higher in the patients with FDG-PET showing lateralization to the surgical side (*n* = 14, Wilcoxon rank sum test, *p* < 0.05). **(C)** SRR was higher in the patients with MSE and FDG-PET showing colateralization to the surgical side (*n* = 14, Wilcoxon rank sum test, *p* < 0.01). *p < 0.05. **p < 0.01. In the box plot, the central mark indicates the median, and the bottom and top edges of the box indicate the 25 and 75th percentiles, respectively. Whiskers extend to the most extreme data points not considered outliers.

A number of ten patients (71%) showed significant differences in averaged MSE scores between hemispheres, and a lower MSE score was assigned to the surgical side in all patients (Tables 1, 2). SRR was higher in the patients with lateralized MSE score (mean SRR: 86.5% and 57.4%, *p* < 0.05) (Figure 1A). Seizure freedom was not associated with the lateralized MSE score (*p* = 0.085). The sensitivity and specificity of the lateralized MSE score to favorable seizure outcome were 88.9 and 60.0%, respectively.

**Table 2:** Averaged multiscale entropy score and FDG-PET lateralization after CC.

**Case**	**Surgical side**	**Averaged MSE score**			**FDG-PET hypometabolic side**	**SRR**
		**L**	**R**	***p-*value**		
1	L	0.46	0.69	<0.001	L	100
2	L	0.92	1.35	<0.001	L	100
3	R	0.54	0.39	<0.05	R	100
4	L	0.31	0.41	<0.05	L	100
5	R	0.81	0.74	<0.01	R	100
6	R	0.51	0.42	<0.01	R	100
7	L	0.52	0.60	0.062	L	95
8	L	0.77	1.06	<0.001	L	95
9	R	0.69	0.41	<0.001	R	95
10	R	0.49	0.48	0.32	R	90
11	L	0.44	0.54	<0.05	L	75
12	R	0.35	0.31	0.074	R	38
13	R	0.25	0.26	0.36	B	0
14	R	0.60	0.56	0.047	L	0

*Significantly decreasing MSE score are underlined. MSE, multiscale entropy; L, left; R, right; SRR, seizure reduction rate (after subsequent surgery)*.

FDG-PET hypometabolism was lateralized to the surgical side in 12 patients (85.7%), and the seizure outcome after subsequent surgery was poor in the remaining two patients with no lateralization (Table 1). SRR was higher in the 12 patients with the lateralized FDG-PET findings (mean SRR: 90.7% and 0%, *p* < 0.05) (Figure 1B). Seizure freedom was not associated with the lateralized FDG-PET findings (*p* = 0.47). The sensitivity and specificity of the lateralized FDG-PET findings to favorable seizure outcome were 100 and 40.0%, respectively.

Multiscale entropy score and FDG-PET hypometabolism were colateralized to the surgical side in 9 patients (64%) (Table 1). SRR was higher in this group (mean SRR: 96.1% and 44.6%, *p* < 0.01) (Figure 1C). Seizure freedom was also associated with colateralized findings (*p* < 0.05). Notably, MSE and FDG-PET were colateralized to the surgical side in all six patients with ILAE class 1 outcome. Colateralization was observed in none of the three patients with ILAE class 5 outcome. The sensitivity and specificity of the colateralized findings to favorable seizure outcome were 88.9 and 80.0%, respectively.

### Representative Cases

#### Case 12

**Figure 2:**
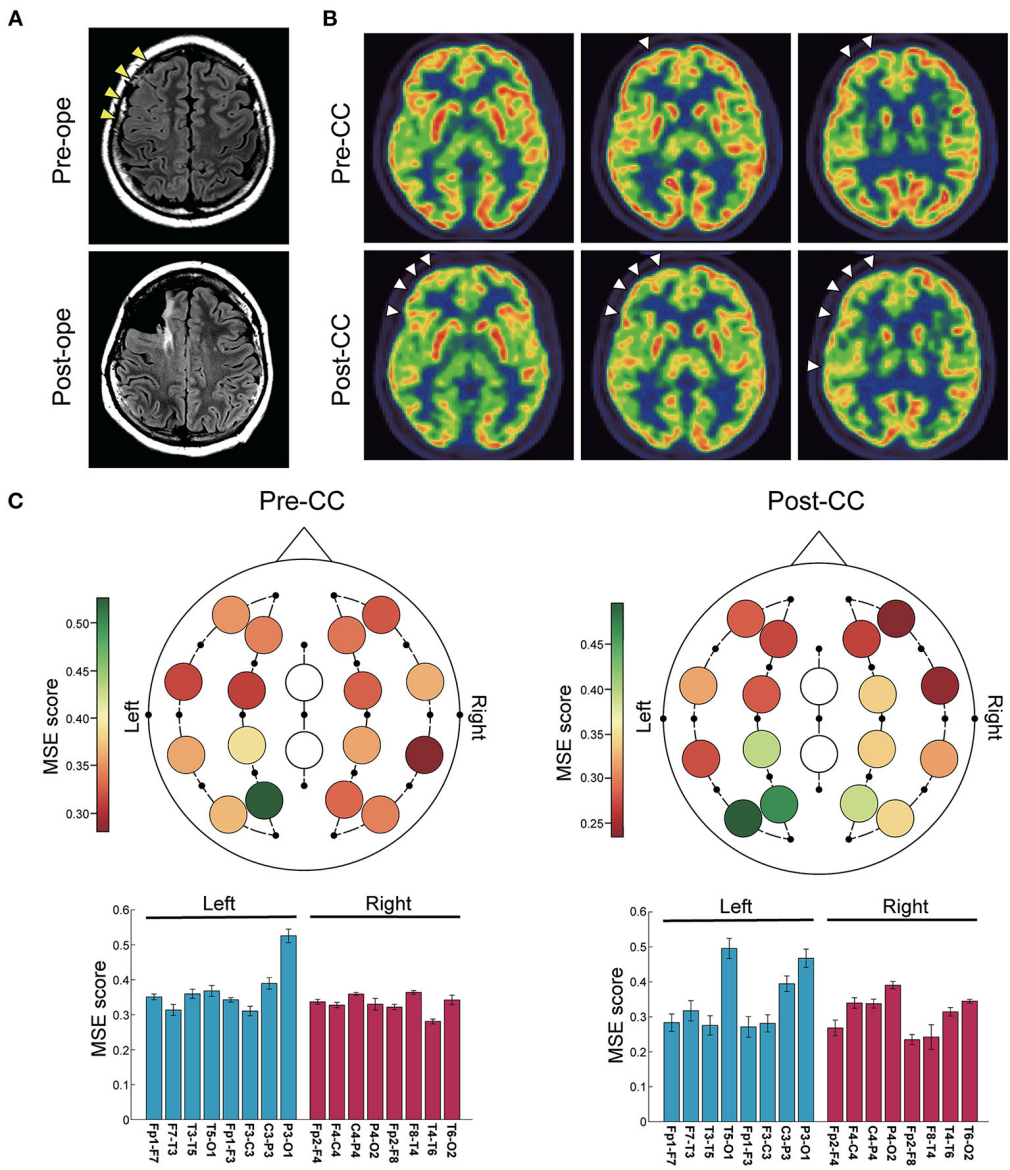
Representative case of a 14-year-old girl with recurrent drop attacks. **(A)** Fluid-attenuated inversion-recovery images showed blurred gray-white matter boundaries in the right frontal lobe. CC was first performed, and after invasive evaluation with intracranial electrode implantation, right frontal lobe FCR was performed. **(B)** Positron emission tomography scans with 2-deoxy-2[^18^F]fluoro-D-glucose showed slight hypometabolism in the right frontal lobe before CC, which became clearer after CC (arrowheads). **(C)** Color maps (upper) and bar plots (lower) of multiscale entropy (MSE) score before and after CC. MSE scores showed no difference between the right and left hemispheres both before and after CC (*n* = 16 channels, two-sided paired *t*-test, before CC: *p* = 0.20; after CC: *p* = 0.074). Bars represent the mean, and lines represent the standard error of the mean.

A 14-year-old girl with daily generalized tonic seizure had bilaterally synchronous epileptic discharges detected by interictal and ictal EEG. Head MRI showed an area with blurred gray-white matter boundaries in the right frontal lobe (Figure 2A), and FDG-PET showed slight hypometabolism in the same area (Figure 2B). However, the patient first underwent CC because the EEG findings were inconsistent with the MRI findings and associated with drop attacks. The drop attacks continued to be intractable even after CC, but repeated examinations revealed that IEDs were lateralized to the right, and ictal EEG suggested a right hemispheric origin. Moreover, the tonicity of the left upper limb became more prominent after CC. After intracranial electrode implantation, FCR was performed on the middle and superior frontal gyri, identified as the origins of the IEDs and ictal EEG onset (Figure 2A). Pathological diagnosis was compatible with focal cortical dysplasia without cytomegalic neurons or balloon cells. MSE score in the left and right hemispheres showed no significant differences both before and after CC (Figure 2C). Although the patient no longer had drop attacks that caused injury, she still had tonic seizures with left head version, and the final seizure outcome was not favorable (SRR: 38%).

#### Case 8

**Figure 3:**
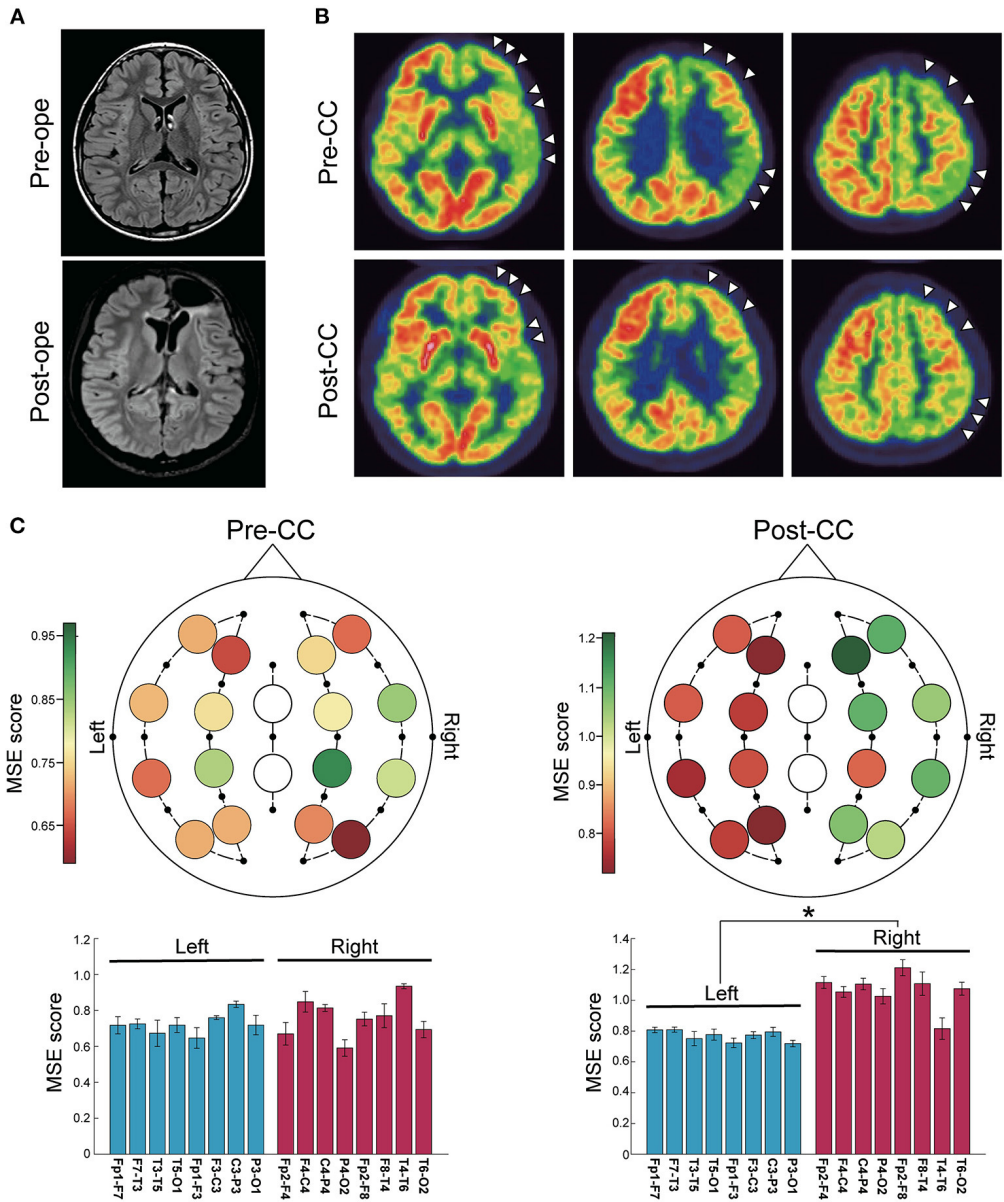
Representative case of a 10-year-old girl with epileptic spasms. **(A)** Fluid-attenuated inversion-recovery images showed no abnormalities. CC was first performed, and after invasive evaluation with intracranial electrode implantation, left frontal lobe FCR was performed. **(B)** Positron emission tomography scans with 2-deoxy-2[^18^F]fluoro-D-glucose showed lateralization to the left hemisphere throughout the pre- and post-CC (arrowheads). **(C)** Color maps (upper) and bar plots (lower) of multiscale entropy (MSE) score before and after CC. MSE score in the left hemisphere was significantly lower than in the right hemisphere after CC (*n* = 16 channels, two-sided paired *t*-test, before CC: *p* = 0.34; after CC: *p* < 0.001). *p < 0.001. Bars represent the mean, and lines represent the standard error of the mean.

A 10-year-old girl with epileptic spasms had bilaterally synchronous IEDs and generalized onset by ictal EEG. Her head MRI showed no abnormalities (Figure 3A), but FDG-PET revealed hypometabolism in the left cerebral hemisphere (Figure 3B). She underwent CC because the resectable epileptogenic zone could not be identified. The seizures continued to be intractable, but IEDs were lateralized to the left, and ictal EEG suggested the origin in the left hemisphere after CC. After intracranial electrode implantation, left frontal lobe FCR was performed (Figure 3A). Post-CC MSE score was significantly lower in the left (Figure 3C). The final seizure outcome was favorable (SRR: 95%).

## Discussion

This study demonstrated that patients with lateralization of MSE and FDG-PET to the surgical side had better outcomes. The combination of two modalities, rather than singly, may be more effective for predicting seizure freedom after resective surgery secondary to CC. Our findings suggested the following two points. First, patients without lateralization of FDG-PET findings are more likely to have poor seizure outcomes and are less likely to be candidates for subsequent surgery after CC. Second, patients with colateralization of MSE and FDG-PET findings are more likely to have good seizure outcomes and are the good candidates for subsequent surgery.

In this study, FDG-PET showed lateralization in 12 of 14 patients, but seizure freedom was achieved only in 6 of 12 patients. The 2 patients without lateralization had the poorest seizure outcomes. Patients with lateralized MSE also had better seizure outcomes but included one patient (Case 14) whose seizures did not improve at all (Table 1). Notably, this case was not associated with FDG-PET lateralization. In addition, FDG-PET had the highest sensitivity at 100%, and the combination of MSE score and FDG-PET had high specificity at 80% to the favorable seizure outcome. In our series, 64% of patients had a favorable seizure outcome (SRR > 95%), similar to the previous report (8). Our findings might suggest how to avoid surgery for patients with predicted poor seizure outcomes.

Based on the mechanism of gamma entropy analysis, our results suggest that interneuron synchronization might be more active during the interictal period in epileptogenic regions than in non-epileptogenic regions, as discussed previously (23, 24). The dynamic activity of inhibitory interneurons is intimately involved in the pathogenesis of focal epilepsy (32–34). Gamma oscillations are dependent on GABA_A_ receptor-mediated inhibition, which suggests that these oscillations are primarily generated by networks of inhibitory interneurons (35, 36). These findings suggest that gamma oscillation in the epileptogenic zone is altered compared to the non-epileptogenic zone, and the synchronization of epileptic interneurons in an epileptogenic zone could be evaluated based on the regularity of gamma oscillation.

There are several limitations of this study. First, only 14 patients are not enough to make generalizations. Due to the small number of patients included in this study, there is heterogeneity in the etiology of epilepsy and the subsequent surgery, which might make the results of the study difficult to interpret. Although we believe that our findings demonstrate the usefulness of MSE on scalp EEG of epilepsy patients, it is necessary to verify the results of this study by accumulating more cases and analyzing a homogenous patient population. Second, in this study, the extraction of EEG data was done manually. It would be desirable to perform automatic and random extraction of EEG data to eliminate potential arbitrariness. Third, FDG-PET results needed to be interpreted with caution because simultaneous EEG was not acquired during the FDG-PET scan. Although we confirmed that there was no obvious seizure at least 1 h before the injection of FDG, it was not denied that subclinical seizure or frequent interictal epileptiform discharges occurred during FDG-PET. Bansal et al. (37) reported that focal hypermetabolism was identified in a large fraction of patients with a spike count of ≥10 per min. Therefore, subclinical seizures and frequent interictal spikes might have influenced FDG-PET results. It is a future issue for us to measure EEG simultaneously with FDG-PET to obtain more interpretable data. Fourth, the spatial resolution of the MSE score was limited because only 16 channels were analyzed. The area with the lowest MSE score did not necessarily correspond to the epileptogenic zone (as described in Figure 3C). Therefore, we only observed the difference in MSE scores between the left and right hemispheres. If more electrodes can be placed in a denser configuration, the spatial value can be expected to increase. Finally, other confounding factors not considered in this study may affect the unfavorable postoperative outcome, such as the absence of focal lesion on MRI (38–40), histologically proven malformation of cortical development (41), and incomplete resection (42). In Cases 8, 9, and 11, seizures persisted even after subsequent surgery despite MSE and FDG-PET were colateralized to the surgical side. Among them, two had no obvious focal lesion on MRI, and it was possible that the extent of resection planned preoperatively was insufficient. Thus, we could not exclude the possibility that the confounding factors mentioned earlier might have influenced the results of this study. Accumulation of more cases may clarify these uncertainties.

## Conclusion

Combination of MSE with conventional FDG-PET may help to select candidates for additional surgery after CC likely to achieve good postoperative seizure outcomes.

## Data Availability Statement

The raw data supporting the conclusions of this article will be made available by the authors, without undue reservation.

## Ethics Statement

The studies involving human participants were reviewed and approved by the Ethics Committees at the National Center of Neurology and Psychiatry in Tokyo, Japan. Written informed consent for participation was not provided by the participants' legal guardians/next of kin because the requirement for written informed consent was waived due to the retrospective design of the study.

## Author Contributions

KK, KI, and MI contributed to study concept, design, and contributed to interpretation of the data. KK and MI contributed to drafting and reviewing the manuscript. All authors contributed to data acquisition and provided final approval for publication.

## Funding

The study was supported, in part, by grants-in-aid for Scientific Research (KAKENHI) grant number JP19K09494 from the Japan Society for the Promotion of Science (JSPS), by the Japan Agency for Medical Research and Development (AMED) under grant numbers JP20ek0109374, JP20ck0106534, JP20he0122002, JP21uk1024005, and JP21wm0425005, and by the Intramural Research Grant (1-4: Integrative research on pathomechanism, diagnostic methodology, and therapeutics for epilepsy) for Neurological and Psychiatric Disorders of the National Center of Neurology and Psychiatry.

## Conflict of Interest

The authors declare that the research was conducted in the absence of any commercial or financial relationships that could be construed as a potential conflict of interest.

## Publisher's Note

All claims expressed in this article are solely those of the authors and do not necessarily represent those of their affiliated organizations, or those of the publisher, the editors and the reviewers. Any product that may be evaluated in this article, or claim that may be made by its manufacturer, is not guaranteed or endorsed by the publisher.

## Abbreviations

CC: Corpus Callosotomy
EEG: Electroencephalography
IED: Interictal Epileptiform Discharge
FDG-PET: positron emission tomography with 2-deoxy-2[^18^F]fluoro-D-glucose
MSE: Multiscale Entropy
MRI: Magnetic Resonance Imaging
ILAE: International League Against Epilepsy
SRR: Seizure Reduction Rate
CT: Computed Tomography.
