# Supplementary material for: Low EEG Gamma Entropy and Glucose Hypometabolism After Corpus Callosotomy Predicts Seizure Outcome After Subsequent Surgery
Source: Front Neurol. 2022 Mar 24;13:831126. doi: 10.3389/fneur.2022.831126 (PMC8989433; doi:10.3389/fneur.2022.831126)

Supplementary Figure 1. Methods for calculating MSE scores for individual channel and comparing MSEs in the left and right hemispheres.

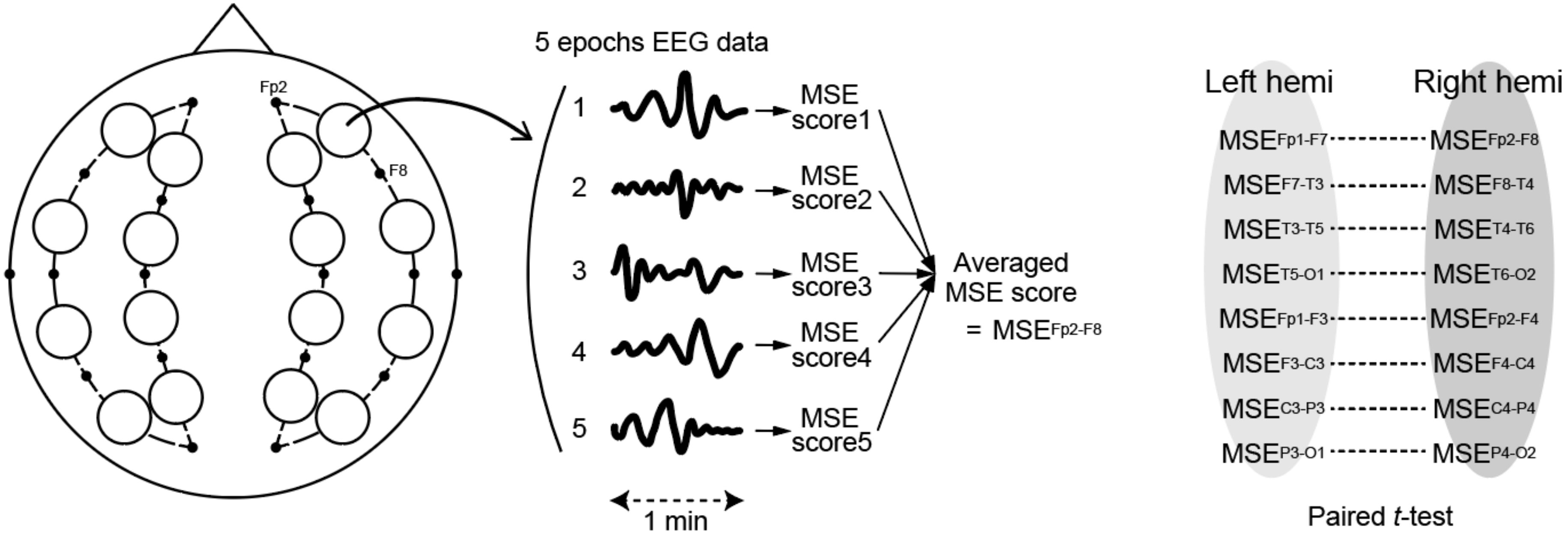

Supplement: Supplementary file 1 [file Image_1.pdf]
