# Supplementary material for: Low EEG Gamma Entropy and Glucose Hypometabolism After Corpus Callosotomy Predicts Seizure Outcome After Subsequent Surgery
Source: Front Neurol. 2022 Mar 24;13:831126. doi: 10.3389/fneur.2022.831126 (PMC8989433; doi:10.3389/fneur.2022.831126)
Supplement: Supplementary file 2 [file Table_1.DOCX]

**Supplementary Table 1.** Summary of presurgical evaluation of corpus callosotomy and postsurgical EEG findings.

| Case | Semiology | pre-CC EEG | post-CC EEG | MEG | MRI | FDG-PET |
| --- | --- | --- | --- | --- | --- | --- |
| 1 | Epileptic spasms | Bilateral temporo-occipital spike | Left middle temporo-occipital spike/Left side onset | Bilateral (temporal and parietal lobe) | Normal | Normal |
| 2 | Epileptic spams | Generalized polyspike | Left fronto-temporo-central spike/ Generalized onset | Left hemisphere | Cortical dysplasia on the left frontal lobe | Hypometabolism on the left frontal lobe |
| 3 | Epileptic spams | Generalized spike | Right frontal/ Generalized onset | Left temporal lobe | Cortical dysplasia on the right frontal lobe | Normal |
| 4 | Epileptic spams | Generalized spike | Left hemispheric spike/ Left side onset | Bilateral (temporal and parietal lobe) | Normal | Normal |
| 5 | Epileptic spams | Multifocal spike | Right hemispheric spike/ Right side onset | Bilateral, left dominant | Right hemispheric slight enlargement | Slight hypometabolism on the right hemisphere |
| 6 | Generalized tonic seizure | Generalized spike | Right cento-parietal spike/ Right side onset | Bilateral (frontal lobe) | Right hemispheric slight atrophy | No exam |
| 7 | Epileptic spams | Multifocal spike | Left hemispheric spike/ Left side onset | Bilateral (temporal and parietal lobe) | Normal | Hypometabolism on the left frontal and parietal lobe |
| 8 | Generalized atonic seizure | Generalized slow spike and wave | Left frontotemporal/Left side onset | Bilateral, right dominant (temporal and parietal lobe) | Normal | Hypometabolism on the left hemisphere |
| 9 | Epileptic spams | Generalized spike | Right hemispheric spike/ Generalized onset | Bilateral (temporal and parietal lobe) | Right hemimegalencephaly | Hypermetabolism on the right hemisphere |
| 10 | Epileptic spams | Generalized spike | Right frontal spike/ Right side onset | Bilateral (temporal and parietal lobe) | Extensive cortical dysplasia on the right hemisphere | Normal |
| 11 | Generalized tonic seizure | Multifocal spike | Left fronto-temporo-central/ Generalized onset | Bilateral (frontal and temporal lobe) | Normal | Hypometabolism on the left temporal lobe |
| 12 | Generalized tonic seizure | Generalized spike | Right frontal spike, generalized spike/ Right side onset | Right frontal lobe | Extensive cortical dysplasia on the right frontal lobe | Hypometabolism on the right frontal lobe |
| 13 | Generalized tonic, atonic seizure | Generalized spike | Right hemispheric/Right side onset | Bilateral (diffuse) | Multiple cortical tubers | Hypometabolism on the left temporal and parietal lobe |
| 14 | Generalized tonic seizure | Generalized spike | Generalized spike, left dominant/ Left side onset | Bilateral (temporal lobe) | Extensive ulegyria on the left hemisphere | Hypometabolism on the left hemisphere |

CC, corpus callosotomy
